# Impaired sweating in patients with cholinergic urticaria is linked to low expression of acetylcholine receptor CHRM3 and acetylcholine esterase in sweat glands

**DOI:** 10.3389/fimmu.2022.955161

**Published:** 2022-07-29

**Authors:** Yiyu Wang, Jörg Scheffel, Carolina Ayala Vera, Wei Liu, Dorothee Günzel, Dorothea Terhorst-Molawi, Marcus Maurer, Sabine Altrichter

**Affiliations:** ^1^ Institute of Allergology, Charité – Universitätsmedizin Berlin, Corporate Member of Freie Universität Berlin and Humboldt-Universität zu Berlin, Berlin, Germany; ^2^ Department of Dermatology, Air Force Medical Center, Beijing, China; ^3^ Fraunhofer Institute for Translational Medicine und Pharmacology (ITMP), Allergology and Immunology, Berlin, Germany; ^4^ Clinical Physiology/Nutritional Medicine, Charité - Universitätsmedizin Berlin, Berlin, Germany; ^5^ Comprehensive Allergy Center, Department of Dermatology and Venerology, Kepler University Hospital, Linz, Austria

**Keywords:** acetylcholine esterase, cholinergic, urticaria, muscarinic 3 receptor, sweat gland, wheal, hypohidrosis/anhidrosis

## Abstract

**Background:**

Cholinergic urticaria (CholU), a frequent form of chronic inducible urticaria, is characterized by itchy wheals and angioedema in response to sweating. As of now, the rate and pathophysiological relevance of impaired sweating in patients with CholU are ill-defined.

**Aim:**

To assess in CholU patients the rate and extent of impaired sweating and its links to clinical and pathophysiological features of CholU.

**Patients and methods:**

We assessed sweating in patients with CholU (*n* = 13) subjected to pulse-controlled ergometry (PCE) provocation testing. Pre- and post-PCE biopsies of lesional (L) and non-lesional (NL) skin were analyzed for the expression of acetylcholine receptor M3 (CHRM3) and acetylcholine esterase (ACh-E) by quantitative histomorphometry and compared to those of healthy control subjects (HCs). CholU patients were assessed for disease duration and severity as well as other clinical features.

**Results:**

Of the 13 patients with CholU, 10 showed reduced sweating in response to PCE provocation, and 3 had severely reduced sweating. Reduced sweating was linked to long disease duration and high disease severity. CholU patients with impaired sweating responses showed reduced sweat gland epithelial expression of CHRM3 and ACh-E.

**Conclusion:**

Reduced sweating is common in CholU patients, especially in those with long-standing and severe disease, and it can be severe. Reduced expression of CHRM3 and ACh-E may be the cause or consequence of CholU in patients with impaired sweating, and this should be explored by further studies.

## Introduction

Cholinergic urticaria (CholU) is one of the most frequent forms of chronic inducible urticaria. The characteristic small itchy wheals are induced by sweating of the patients. Triggers can be physical activity and passive warming but also hot or spicy food and emotional stress (1). This disease most frequently starts in late puberty and affects young adults, with up to 20% in some studies being affected (2), although the disease can also start later in life, predominantly in women (3).

The term “cholinergic” is derived from the finding that hives similar to those of CholU can be evoked using cholinergic agonists (e.g., methacholine, acetylcholine) in skin tests (4–6). To date, the understanding of the pathomechanism of CholU is incomplete and several different concepts had been developed. However, it is common in all theories that the sweating process plays a central role. The pathomechanistic concepts proposed for the disease (7, 8) include i) blockage of the sweat gland duct and induction of symptoms by sweat leakage into the tissue (9); ii) IgE-mediated mast cell activation induced by sweat antigen(s) (10), supported by the fact that therapy with the anti-IgE antibody omalizumab can be beneficial in CholU patients (11–13); and iii) association with reduced sweating (hypohidrosis) or complete lack of sweating (anhidrosis) in at least some body areas (8, 14, 15). In this last group of patients, the destruction of the sweat glands by autoimmune mechanisms had been proposed as an underlying process and was supported by clinical cases where immunosuppressive therapies using corticosteroids proved to be beneficial. In other patients, aberrations in the sympathetic neuron–sweat gland interaction had been proposed as the underlying pathophysiological problem (16).

In the past, studies have shown that CholU patients with hypohidrosis or anhidrosis develop wheals in the hypohidrotic areas of the skin. Biopsies from these skin areas showed decreased expression of the acetylcholine receptor M3 (CHRM3) of the sweat glands (17, 18). Furthermore, reduced expression of the Ach-degrading enzyme acetylcholine esterase (ACh-E) in hypohidrotic areas of CholU patients was seen (18, 19). As a consequence, it was speculated that acetylcholine (ACh) released from the sympathetic nerves to induce sweat production at the sweat glands cannot be completely trapped by acetylcholine receptors of the eccrine glands. The subsequent overflow of ACh to the adjacent mast cells then leads to their degranulation, possibly mediated *via* CHRM3 receptors (18, 20, 21).

Most studies and patient reports dealing with this topic more recently had been studying Japanese patients (22). To our knowledge, no detailed investigation of the sweating behavior of Caucasian CholU patients and its link to clinical and histological features has been published. Accordingly, this study aims at investigating the sweating behavior of a cohort of well-characterized CholU patients and 1) to link sweating ability to clinical features of the patients, 2) to investigate a possible correlation with the expression of the ACh receptor CHRM3 and the Ach-degrading enzyme ACh-E in the same patients, and 3) to investigate the possible underlying pathomechanisms for the observed aberrations.

## Materials and methods

### Study subjects

In this study, 13 patients with CholU were recruited at the Urticaria Center of and Excellence (UCARE) (23) of the Department of Dermatology and Allergy, Charité - Universitätsmedizin Berlin for provocation testing and extensive clinical characterization. After informed consent, patients were advised to stop antihistamine intake at least 3 days before any of the mentioned tests below. None of the patients had been taking local or systemic steroids or other immunosuppressive therapy in the last 2 weeks before the tests. Twelve matched healthy volunteers who underwent the same procedures served as the control group.

The demographic characteristics of the study participants are shown in **Supplementary Table 1**. This study was approved by the Ethics Committee of the Charité - Universitätsmedizin Berlin (#EA1/241/15) and registered in the German Clinical Trials Register (DRKS-ID: DRKS00012755).

### Clinical assessments

Twelve healthy controls and 13 patients with CholU were assessed for clinical history and comorbidities, as well as atopic skin diathesis (atopic predisposition, by using the Erlangen Atopy Skin Score (24)). The Erlanger criteria for the assessment of an atopic skin diathesis were developed to analyze, independently of the current skin disease, the atopic diathesis with respect to the target organ skin, including assessment of atopic diseases in the past and present and scoring minimal atopic stigmata.

CholU patients were assessed for their disease duration and disease severity during the last 2 weeks *via* a visual analog scale (VAS) ranging from 0 to 10 and a four-dimensional Likert scale (0 = no, 1 = mild, 2 = moderate, 3 = severe symptoms). Furthermore, patients rated their overall disease severity with the Cholinergic Urticaria Severity Index (CholUSI), a sum score that takes into account the frequency of CholU symptoms, eliciting factors, duration of skin lesions, and itch (25). The CholUSI score ranges from 0 to 21 points, with higher scores reflecting higher disease severity.

CholU-specific disease activity was assessed from the last 7 days before presentation using the cholinergic urticaria activity score (CholUAS7; MOXIE GmbH, Berlin, Germany) (26). This score assesses the number of wheals and intensity of itching—each on a 0–3 scale each day—concerning the exposure trigger. The CholUAS was recorded by each patient daily, and a weekly sum score was obtained from the patients. The higher the daily and weekly score, the more active the disease was at that time point.

Quality of life impairment was evaluated using the general usable Dermatology Life Quality Index (DLQI) (27), which has a 1-week recall period, and the disease-specific Cholinergic Urticaria Quality of Life Questionnaire (CholU-QoL; MOXIE GmbH) (28), which has a 2-week recall period. Higher scorings reflect a higher quality of life impairment.

Disease control was assessed with the urticaria control test (UCT; MOXIE GmbH), a four-item questionnaire (29). Disease control is achieved if patients reach 12 points or more.

### Pulse-controlled ergometry test

In all participants (*n* = 13), pulse-controlled ergometry (PCE) testing was performed as described before (25). Briefly, patients underwent physical exercise on a stationary bike with increasing intensity over a time period of 30 min reaching a maximal pulse rate of about 160 beats per minute. Patients were assessed for the time to onset of sweating using the iodine–starch reaction (test according to Minor (30)), the time to onset of whealing, and the increase in heart rate at the onset of whealing.

Furthermore, patients were assessed for their symptoms at the end of the provocation test (itching: no itch = 0, mild itch = 1, moderate itch = 2, severe itch = 3; whealing: no whealing = 0, 1–20 wheals = 1, 21–50 wheals = 2, >50 wheals = 3) resulting in the sum score ProvoUAS (0–6 points). Also, at the end of the provocation test, the amount of sweating was assessed and patients were grouped into three categories: severely reduced sweating (SRS), i.e., minimal black/blue dots; reduced sweating (RS), i.e., less than half of the area is black/blue; or normal sweating (NS), i.e., more than half of the area is black/blue as assessed by the use of the iodine–starch test according to Minor (see **Supplementary Figure 1**).

### Skin biopsy/histology

We obtained 5-mm full-thickness skin biopsies before (non-lesional skin) and 10–15 min after the end of the PCE test from both CholU patients (lesional skin) and HCs (non-lesional skin). The lesional skin biopsies in CholU patients were taken from emerging wheals right after the PCE test. Typically, biopsies were taken on the upper arm or trunk of the participants in close proximity to each other of no more than 20-cm distance. Skin specimens were immediately fixed in 4% buffered formaldehyde (Herbeta Arzneimittel, Berlin, Germany) for at least 24 h. The embedding of the section in paraffin was performed using the Shandon Citadel 1000 (Waltham, USA) using ethanol for dehydration (up to 99%; Merck, Darmstadt, Germany), followed by xylene (Merck) immersion and paraffin embedding. After embedding, 5-μm-thick tissue sections were cut using a Microtome Finesse 325 from Shandon (Waltham, USA). After paraffin removal with xylene and ethanol, slides were heated at 99°C–100°C in 0.01 M of sodium citrate buffer, pH 6.0 for antigen retrieval for 20 min. After washing with Tris-buffered saline with 0.05% Tween 20 (TBS-T), the slides were blocked with universal protein block (Cat. #X0909, Dako, Carpinteria, CA, USA) for 20 min at room temperature.

These sections were first stained with rabbit anti-human acetylcholine esterase antibody (Cat. #LS-B6676, Lifespan Biosciences, Germany) or rabbit anti-human CHRM3/M3 antibody (Cat. #LS-C144026, Lifespan Biosciences) diluted 1:90 in TBS-T buffer for 60 min at room temperature. After TBS-T washings, a directly labeled polymer-AP anti-rabbit antibody (Cat. #MP-5401, Vector Laboratories, Burlingame, CA, USA) was used for detection. Immunohistochemical staining was performed using alkaline phosphatase chromogen substrate (Cat. #SK-5100, Vector Laboratories). The slides were mounted with Aquatex^®^ mounting medium (Cat. #108562, Merck) and stored in the dark at room temperature until analysis.

For immunofluorescence imaging, a goat anti-rabbit antibody conjugated to Alexa Fluor 488 or Alexa Fluor 594 (Cat. #111-545-144 or Cat. #111-585-144, Jackson ImmunoResearch, PA, USA) at a concentration of 3 µg/ml was used as a secondary antibody. The slides were mounted with Fluoromount-G Mounting Medium containing DAPI for nuclear staining (Cat. #00-4959-52, Invitrogen, Carlsbad, CA, USA) and stored in the dark at room temperature until analysis.

The sections were analyzed using the Axioplan2 microscope (Zeiss, Oberkochen, Germany). On average, 25 high-power fields (×40 objective, ×10/20 eyepiece) corresponding to 1.25 mm^2^ were assessed. Counting of positive cells and staining intensity analysis were performed using ImageJ software (open source, https://imagej.nih.gov/ij/download.html, NIH, USA). Mean staining intensity was calculated as described in (31, 32) and was given as “red density.” In brief, histograms revealed 255 different shades from pitch black (0) to pure white (255), and a numerical value (units) represented the level of brightness of each color. We analyzed the mean intensity of the histogram of shades that represent the red color spectrum (since the immunostainings resulted in a red color) in the cytoplasm and averaged the value of three different areas. To obtain the red density, we calculated the mean intensity of each color.

### IgE measurements

Total and specific IgE levels were measured at the central laboratory (Labor Berlin GmbH, Berlin, Germany) using the ImmunoCAP System (Phadia Laboratory Systems, Thermo Fisher Scientific Inc., Uppsala, Sweden). Total IgE levels >100 kU/L were considered elevated.

### Statistical analyses

Statistical analyses were performed using IBM SPSS Statistics version 23 and GraphPad Prism Version 6.0. Binominal variables were analyzed using the chi-square test or Fisher’s exact test for low numbers. Mean values were compared using the Student’s *t*-test for two groups or the Kruskal–Wallis test for the comparison of more than two groups. Correlations were analyzed using the Spearman rank test. *p <*0.05 was considered to indicate statistical significance.

## Results

### In CholU, impaired sweating is common and linked to long disease persistence and high disease severity

**Figure 1:**
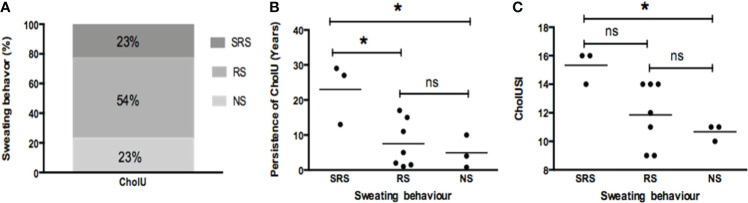
**(A)** More than half of cholinergic urticaria (CholU) patients showed reduced sweating (RS); the remaining had either normal sweating (NS) or severely reduced sweating (SRS). CholU patients grouped *via* their sweating behavior show differences regarding **(B)** persistence of disease and **(C)** severity of disease (assessed by CholUSI). *p < 0.05; ns, not significant.

Ten of 13 patients with CholU (77%) showed reduced (7/13, 54%) or severely reduced sweating (3/13, 23%) in response to PCE provocation testing (**Figure 1A** and **Supplementary Figure 1**). Sweating was reduced or severely reduced in eight of nine (89%) male and two of four (50%) female CholU patients. All healthy controls (nine men, four women) showed normal sweating behavior (data not shown).

CholU patients with severely reduced sweating showed significantly longer persistence of disease (mean ± SD: 23 ± 8.7 years) as compared to patients with reduced sweating (7.5 ± 6.7 years; *p* = 0.02) or normal sweating responses (8.0 ± 3.4 years; *p* = 0.05; **Figure 1B**). Disease severity, as assessed by the CholUSI, was the highest in CholU patients with severely impaired sweating, followed by patients with reduced sweating, and the lowest in those with normal seating responses (**Figure 1C**).

Impaired sweating in patients with CholU was not linked to age at disease onset, disease activity as assessed by the CholUAS7 or ProvoUAS, quality of life impairment (DLQI, CholU-Qol), disease control (UCT), the Erlangen Atopy score, or serum levels of total IgE, although the latter were numerically higher in patients with severely impaired sweating (391.4 ± 383.5 IU/ml) as compared to those with reduced and normal sweating responses (122.5 ± 110.3 and 147 ± 57.2 IU/ml, respectively; **Supplementary Table 2**).

### Sweat gland expression of the acetylcholine receptor CHRM3 is reduced in patients with CholU, especially in those with severely impaired sweating responses

**Figure 2:**
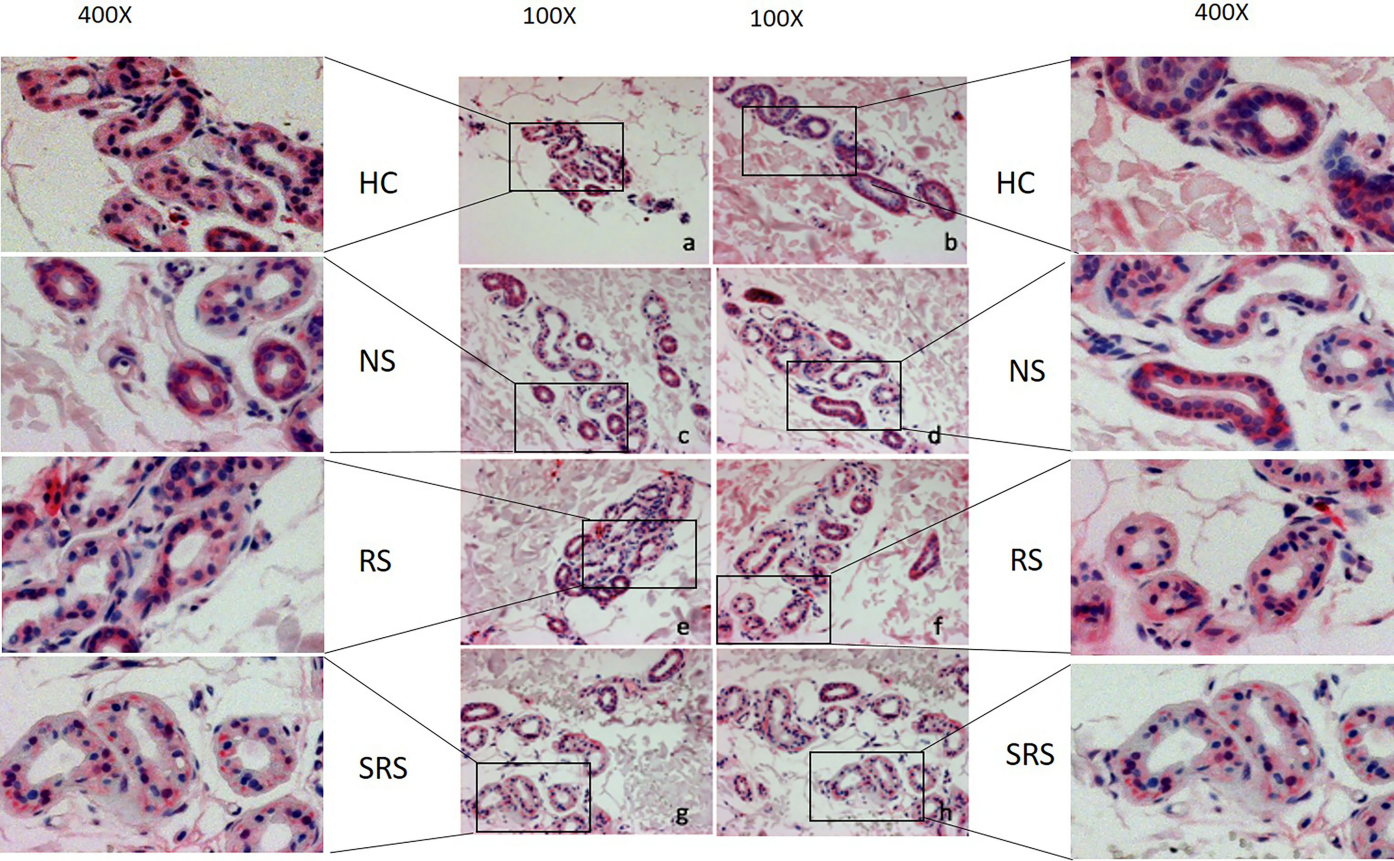
Representative immunohistochemical examination patterns of the expression of CHRM3 of HC and CholU patients separated per sweating group: **(a/ c/ e/ g)** non-lesional skin samples taken before provocation, **(b)** non-lesional skin after provocation from a sample of the HC group, and **(d/ f/ h)** lesional skin samples after provocation from CholU patients. Zoom-in boxes depict the cutouts in higher magnifications. HC, healthy controls; NS, normal sweating CholU; RS, reduced sweating CholU; SRS, severely reduced sweating CholU.

**Figure 3:**
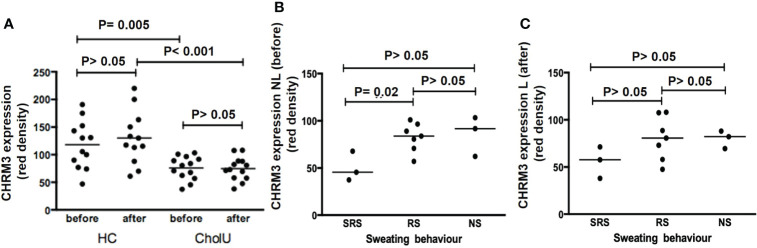
**(A)** CHRM3 expression on the sweat gland epithelium of HC and CholU patients’ skin before and after pulse-controlled ergometry (PCE). **(B)** CHRM3 expression within CholU patients was significantly reduced before PCE provocation in the SRS patient group. **(C)** A similar trend was seen in the skin samples after provocation, but here no significance level was reached. NS, normal sweating CholU; RS, reduced sweating CholU; SRS, severely reduced sweating CholU.

As compared to the sweat glands of healthy control skin, which showed strong homogeneous expression of the ACh CHRM3, the sweat glands of CholU patients exhibited patchy and significantly lower CHRM3 expression, as assessed by quantitative histomorphometry (76 ± 21 vs. 118 ± 43 U, *p* = 0.0047, **Figures 2**, **3**).

PCE provocation increased sweat gland CHMR3 expression in healthy control subjects, albeit not significantly, from 118 ± 43 to 130 ± 48 U. In contrast, CHRM3 expression after PCE was unchanged in CholU patient samples (76 ± 21 vs.75 ± 21 U; **Figure 3A**).

In CholU patients, sweat gland CHRM3 expression was the lowest in those with severely impaired sweating responses, both in non-lesional and lesional skin (**Figures 3B, C** **)**.

### Sweat gland expression of acetylcholine esterase is reduced in patients with CholU, especially in those with severely impaired sweating responses

**Figure 4:**
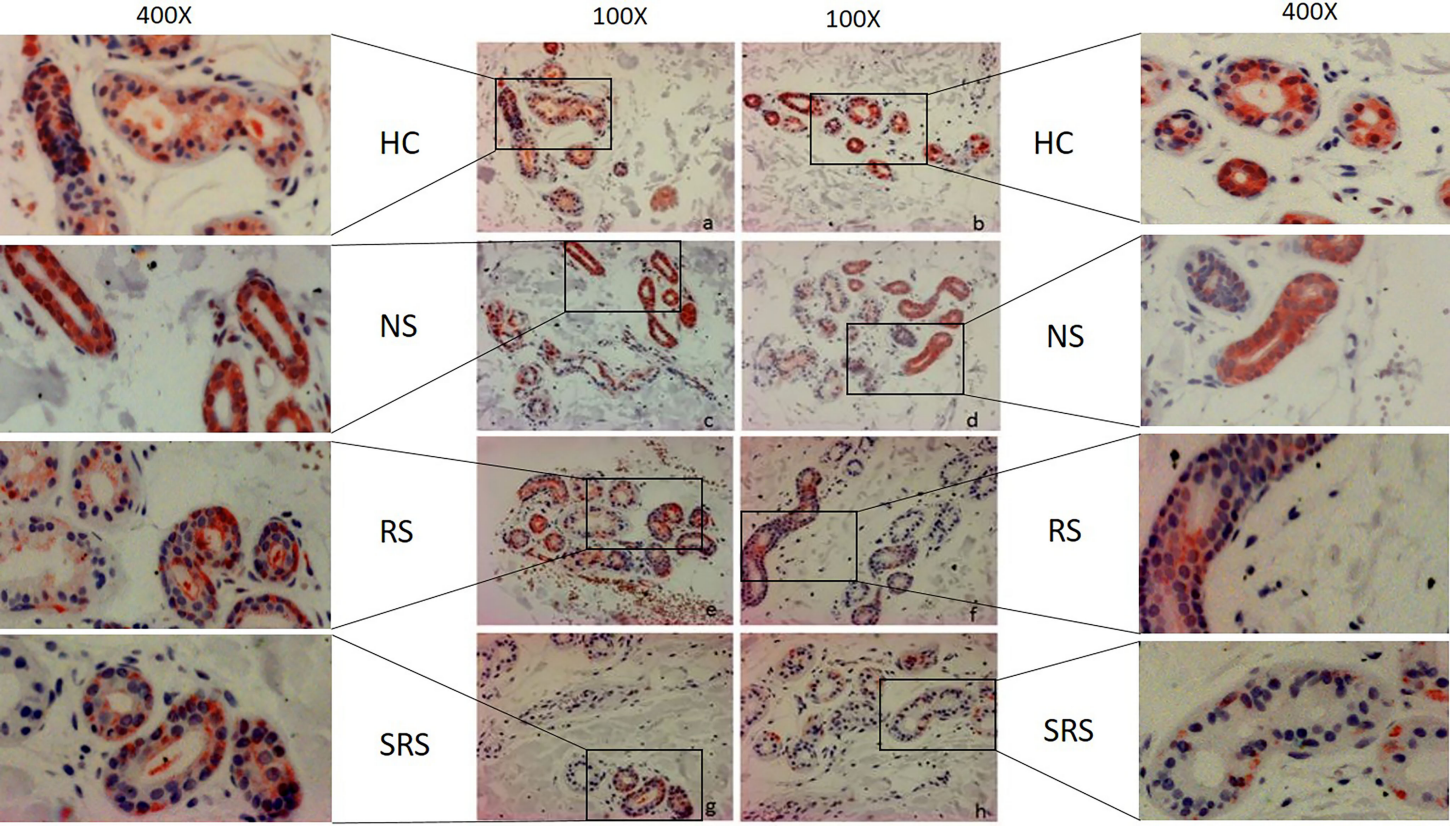
Representative immunohistochemical examination patterns of the expression of ACh-E of HC and CholU patients separated per sweating group: **a/ c/ e/ g/** non-lesional skin samples taken before provocation, **b** non-lesion after provocation from the HC group, and **d/ f/ h** lesional skin samples after provocation from CholU patients. Zoom-in boxes depict the cutouts in higher magnifications. HC, healthy controls; NS, normal sweating CholU; RS, reduced sweating CholU; SRS, severely reduced sweating CholU.

**Figure 5:**
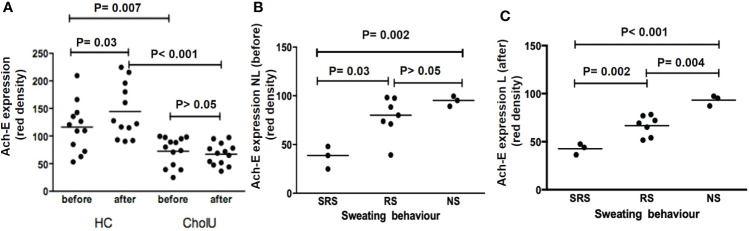
**(A)** ACh-E expression on sweat gland epithelium in HC and CholU patients’ skin before and after PCE. **(B)** ACh-E expression within CholU patients was significantly reduced before PCE provocation in the SRS patient group. **(C)** Significant differences were seen between all sweating groups in CholU patients with high significance. HC, healthy controls; NS, normal sweating CholU; RS, reduced sweating CholU; SRS, severely reduced sweating CholU.

Although the sweat glands of healthy control skin showed strong homogeneous expression of the ACh-degrading enzyme ACh-E, CholU patients exhibited heterogeneous and markedly reduced ACh-E expression as assessed by quantitative histomorphometry, i.e., 73 ± 26 vs. 116 ± 45 U (*p* = 0.007; **Figures 4**, **5**).

PCE provocation increased sweat gland ACh-E expression in healthy control subjects, from 116 ± 45 to 144 ± 49 U (*p* = 0.03). In contrast, ACh-E expression before PCE in non-lesional skin and lesional skin after PCE was unchanged in the sweat glands of CholU patients (73 ± 26 vs. 67 ± 20 U; *p* > 0.05; **Figure 5A**).

In CholU patients, sweat gland ACh-E expression was the lowest in those with severely impaired sweating responses, both in non-lesional and lesional skin (**Figures 5B, C** **)**.

### In patients with CholU, the expression of sweat gland CHRM3 and ACh-E is closely correlated and the latter is linked to disease activity and duration

**Figure 6:**
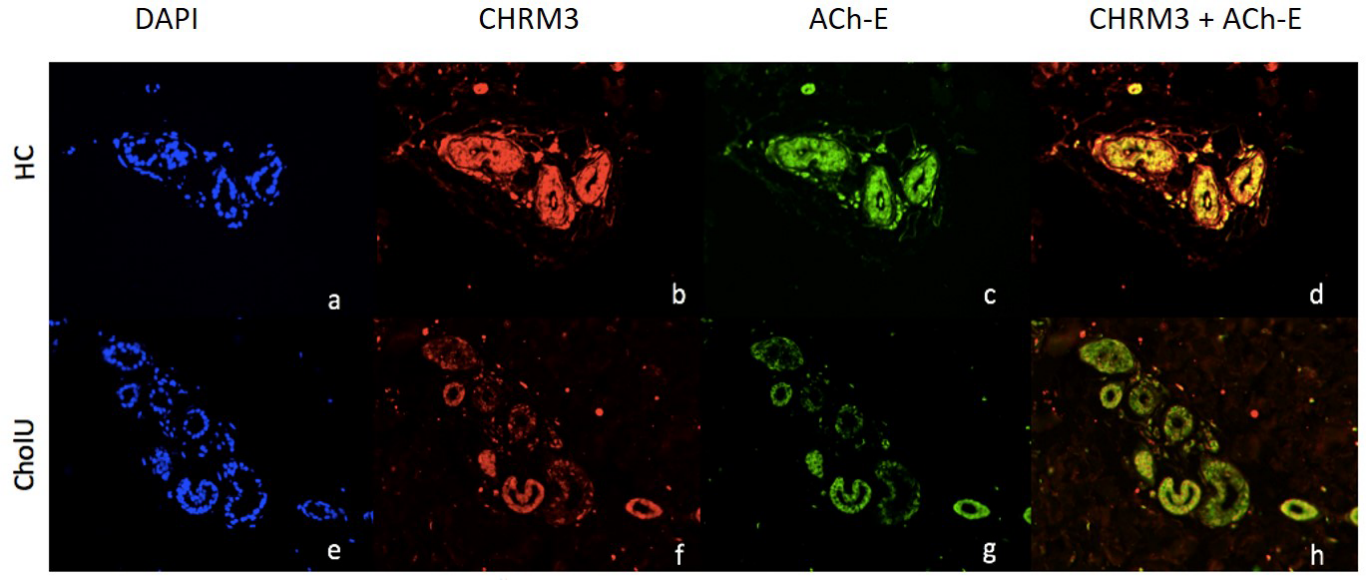
Representative immunohistochemical examination patterns of the expression of CHRM3 and ACh-E of non-lesional skin before provocation in HC **(A–D)** and CholU patients **(E–H)**. **(A, D)** DAPI staining of nuclei of sweat glands; **(B, E)** CHRM3 staining (red); **(C, F)** ACh-E staining (FITC) d/g CHRM3 and ACh-E overlay.

In patients with CholU, sweat gland CHRM3 and ACh-E were co-expressed, with a striking overlap of areas of low expression of both (**Figure 6**). The expression levels of CholU sweat gland CHRM3 and ACh-E were strongly correlated in both lesional (*r* = 0.90, *p* < 0.001) and non-lesional skin (*r* = 0.73, *p* < 0.001).

The expression levels of sweat gland ACh-E, but not CHRM3, in lesional and non-lesional skin were strongly linked to CholU duration and severity. The lower the expression, the longer (NL: *r* = −0.69, *p* = 0.009; L: *r* = −0.77, *p* = 0.002) and the more severe the disease (NL: *r* = −0.63, *p* = 0.02; L: *r* = −0.58, *p* = 0.04) as assessed by CholUSI (see **Supplementary Table 3**).

## Discussion

This study of CholU patients from a specialized urticaria center in Germany showed for the first time that sweating impairment (hypohidrosis) is a common feature also in these Caucasian patients since most of the reports on sweating impairment in CholU in the literature come from Asian countries (8). In our cohort, one-fourth of the patients displayed severely reduced sweating in the tested area on the trunk, but none had a complete generalized anhidrosis. Of note, most patients did not report impaired sweating, as they showed good or even exaggerated sweating in the axillary area, face, hands, and feet. Rho et al. (15) reported in a retrospective analysis that 7% of the CholU patients from a military hospital in Korea complained of sweating abnormalities. The authors also reported normal sweating in the axillae, palms, popliteal fossae, and plantar surfaces of these patients. Possibly, this rate would have been higher if the patients had been systematically assessed for local sweating abnormalities, as we experienced that not all patients are aware of their sweating impairment.

In our patient cohort, sweating impairment seemed to be more prevalent in men than in women; however, the number of study participants was too small to make a reliable statement. Nevertheless, Japanese authors also reported more often male than female patients with CholU-associated hypohidrosis (9, 18, 33). Differences with respect to sex have also been seen during the onset of disease (3) and also with respect to treatment responses (34), suggesting that hormonal or other sexual differences could play an important role in the pathophysiology of the disease.

Usually, CholU is a disease that would start in puberty or early adolescence, although late onset is also possible (3). CholU usually persists for several years up to one or two decades before it will resolve without sequelae. In this study, the disease persistence in our cohort ranged from 1 to 29 years, with the longest persistence in the SRS group. Aside from the long persistence, a trend toward higher disease severity in the CholUSI has been seen, which would fit case reports reporting high disease burden and severe symptoms in this patient group as well as low effectiveness of antihistamine treatment (9, 15, 17, 20).

ACh is the driving neurotransmitter that upon binding to the CHMR3 receptor on the sweat gland secretory cells as well as in myoepithelial cells induces sweat production in humans (35). This suggests that impaired sweating in CholU is due to reduced CHRM3 expression, at least in part. In our study, we saw an overall strongly and heterogeneously patchy reduced CHRM3 expression in CholU patients with SRS capability. The lowered expression is in line with previous reports, where binding assays were used to show that muscarinic cholinergic receptors are reduced in the skin of patients with CholU and hypohidrosis as compared to healthy controls (31). Sawada et al. also showed that CholU patients develop wheals exclusively in the hypohidrotic area where the expression of CHRM3 is incompletely decreased in sweat gland epithelial cells and mast cells, whereas the patients did not exhibit wheals in the anhidrotic area where CHRM3 expression is completely absent (31). Our samples were taken from areas where patients do develop wheals, and none of our samples had completely absent CHRM3 expression, confirming this previous observation. Since we have not assessed the total body sweating capacity, we cannot rule out that patients do also have anhidrotic areas, but patient interrogations did not suggest this.

ACh-E is a degrading enzyme of ACh, which breaks down ACh once internalized together with the receptor into the sweat gland cell, limiting or stopping sweat production (36). Downregulation of this enzyme could be a specific feature of CholU as suggested before (18).

ACh is known to induce whealing when injected intradermally into the skin, at least in some CholU patients (37). Accordingly, current theories suggest that the excess of ACh, which might not be able to bind to the receptor and get degraded, leads to a direct mast cell activation (38, 39), which in turn induces the wheal and flare response in these patients.

In contrast to prior publications (18, 31), we did not see an increased cellular infiltration around the sweat glands. Furthermore, no visible destruction of the sweat gland epithelium, which could explain the reduced expression, was visible.

We observed a strong co-localization of CHRM3 and ACh-E in healthy controls. In patients, however, CHRM3 and ACh-E expression levels were both downregulated. More prominent was the ACh-E downregulation, compared to the CHRM3 downregulation, beeing associated with the clinical picture of long disease persistence and severe disease seen in SRS patients. The factors which cause this downregulation are currently unknown. We think that there could be two main pathophysiological reasons for this strong correlation: i) If there are not enough CHRM3 receptors expressed, ACh-E is compensatory-downregulated (secondarily) to keep ACh levels up in the affected areas or there is less need for this degrading enzyme; ii) or the other way around, if the ACh-E is too low, as a compensation, the CHRM3 receptors are downregulated to avoid excess ACh stimulation of the sweat glands.

The histological analysis revealed a patchy picture within individual cross-sections of the sweat gland ducts. Overall, this points to a localized process rather than a general systemic influence that causes the phenomenon. Furthermore, not all sweat glands are impaired in the same way, since sweating is not impaired in special sites like the armpits, face, hands, and soles (14, 15). Why some parts of the sweat glands or some areas are affected by the downregulation, whereas others seem normal, is currently unknown.

Beyond sweating, CHRM3 and ACh-E are important key factors for neurological functions such as REM sleep (40) and adequate cognition (41), and anticholinergic drugs had been associated with an increased risk of dementia (42). If patients with CholU have impaired neurological functions or have a higher risk to develop impairments in these areas is currently unknown. A specific test would be needed to systematically assess patients. More recently, new therapeutic concepts for CholU that focus on hypohidrosis have been reported (33). Regular sweating activities for the treatment of cholinergic urticarial were applied to CholU patients with sweating impairment (~50%) and without sweating impairment. In the sweating therapy group, complete response was achieved by six of the eight (75%) patients without acquired idiopathic generalized anhidrosis and two of the four (50%) patients with acquired idiopathic generalized anhidrosis. How the mechanism of repetitive sweating treatment works is not entirely clear. It seems possible that regular sweating improves the CHRM3 and ACh-E expression and, by this, reduces the sweating-associated symptoms. On the other hand, regular sweating could induce desensitization *via* a direct mast cell-targeted mechanism, similar to what had been described, e.g., for drug hypersensitivity and desensitization (43), where antigen-specific processes lead to blockings of the calcium flux cascades and impacts antigen/IgE/FcεRI complex internalisation. This would be supported by studies that reported improvement of symptoms after a specific immunotherapy with purified sweat antigen (44). That mast cells indeed have an important role in CholU was recently confirmed in a study with lirentelimab, a drug that directly targets mast cell function, which leads to a good improvement in almost all of the patients in a small proof-of-concept study (45).

The limitations of this study are mainly due to a possible selection of patients in a tertiary specialized center and the relatively low number of CholU patients that were assessed. The later subgrouping of the patients according to their sweating behavior then leads to very low individual numbers in each group. Nevertheless, the patients were intensely examined including skin biopsies. In this respect, it is one of the biggest studies regarding this topic so far. Another limitation is the localized Minor’s sweat test on the back, which does not allow the identification of sweating impairment elsewhere. The limitations in the interpretation of the histological analysis are that we cannot rule out that in different biopsy samples different proportions of acini and ducts in the sections could contribute to the differences seen between the groups.

In summary, in this study, we could assert that reduced sweating is common, also in Caucasian CholU patients, including a small subgroup with severely reduced sweating behavior with long disease persistence and higher disease severity. This reduced sweating showed a strong link to the ACh-mediated nerve–sweat gland interaction mechanism including reduced CHRM3 and ACh-E expression, as confirmed in this study. The cause of this dysregulation remains elusive and should be investigated in further studies.

## Data availability statement

The raw data supporting the conclusions of this article will be made available by the authors, without undue reservation.

## Ethics statement

The studies involving human participants were reviewed and approved by ethics board Charite-Universitätsmedizin Berlin. The patients/participants provided their written informed consent to participate in this study.

## Author contributions

YW analyzed the samples and was involved in statistical analysis, manuscript preparation, and proofreading of the manuscript. JS supervised the sample analysis and was involved in the statistical analysis and proofreading of the manuscript. CV was involved in the sample analysis and proofreading of the manuscript. WL was involved in the proofreading of the manuscript. DG was involved in result interpretation and critical proofreading of the manuscript. DT-M was involved in the statistical analysis and proofreading of the manuscript. MM was the overall study coordinator and was involved in manuscript preparation and proofreading of the manuscript. SA coordinated the study, collected the patient data, was involved in the statistical analysis, and drafted the manuscript. All authors contributed to the article and approved the submitted version.

## Acknowledgments

We want to thank Evelin Hagen for her excellent support in the sample analysis and Beate Schinzel for the assistance.

## Conflict of interest

DT-M has received research funds/was an advisor for Celldex, Novartis, Sanofi, and Moxie. MM is or recently was a speaker and/or advisor for and/or has received research funding from Allakos, Amgen, Aralez, ArgenX, AstraZeneca, Celldex, Centogene, CSL Behring, FAES, Genentech, GIInnovation, Innate Pharma, Kyowa Kirin, Leo Pharma, Lilly, Menarini, Moxie, Novartis, Roche, Sanofi/Regeneron, Third HarmonicBio, UCB, and Uriach. SA has been a speaker and/or advisor for and/or has conducted studies for AstraZeneca, Allakos, GSK, Leo Pharma, Lilly, Moxie, Novartis, Thermo Fisher, and Sanofi.

The remaining authors declare that the research was conducted in the absence of any commercial or financial relationships that could be construed as a potential conflict of interest.

## Publisher’s note

All claims expressed in this article are solely those of the authors and do not necessarily represent those of their affiliated organizations, or those of the publisher, the editors and the reviewers. Any product that may be evaluated in this article, or claim that may be made by its manufacturer, is not guaranteed or endorsed by the publisher.

## Abbreviations

Ach, acetylcholine; ACh-E, acetylcholine esterase; CholU, cholinergic urticaria; CHRM3, acetylcholine receptor M3; CIndU, chronic inducible urticaria; CSU, chronic spontaneous urticaria; U, units; wks, weeks; y, years.
