# Supplementary figures and images for: Impaired sweating in patients with cholinergic urticaria is linked to low expression of acetylcholine receptor CHRM3 and acetylcholine esterase in sweat glands
Source: Front Immunol. 2022 Jul 29;13:955161. doi: 10.3389/fimmu.2022.955161 (PMC9373796; doi:10.3389/fimmu.2022.955161)

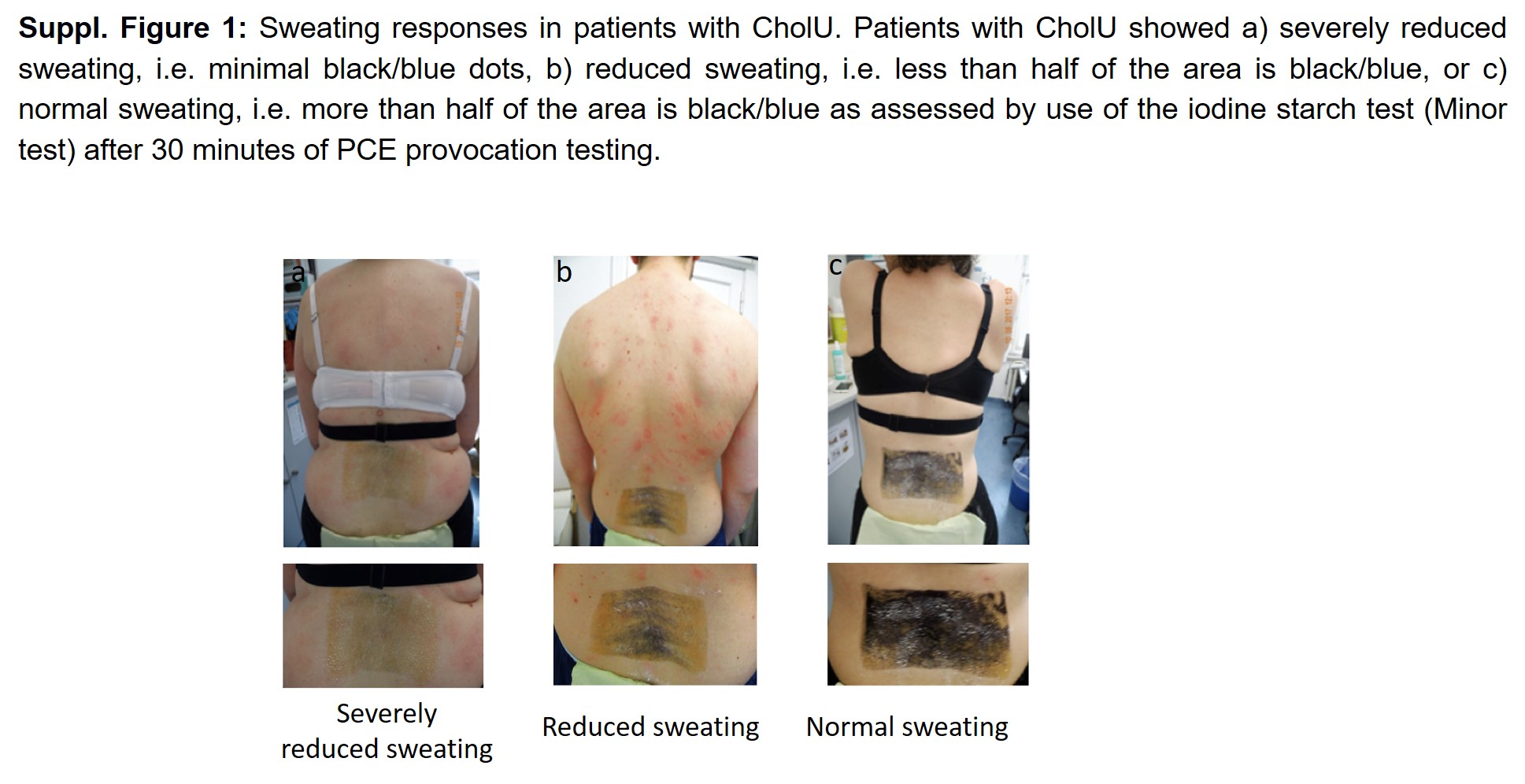

Supplement: Supplementary file 1 [file Image_1.jpeg]
