# Supplementary material for: Impaired sweating in patients with cholinergic urticaria is linked to low expression of acetylcholine receptor CHRM3 and acetylcholine esterase in sweat glands
Source: Front Immunol. 2022 Jul 29;13:955161. doi: 10.3389/fimmu.2022.955161 (PMC9373796; doi:10.3389/fimmu.2022.955161)
Supplement: Supplementary file 2 [file Table_1.docx]

**Table S1.** Participant demographics

| **Parameter** | **Healthy controls** | **CholU patients** | **p** |
| --- | --- | --- | --- |
| **Number** | 12 | 13 |  |
| **Sex (m/f)** | 8/4 | 9/4 | > 0.05 |
| **Age (years)**  mean ± SD  median (IQR) | 34.6 ± 13.8  33 (22.5 – 43.3) | 33.5 ± 11.1  31 (24 - 42) | > 0.05 |
| **Body mass index (kg/m^2^)**  mean ± SD  median (IQR) | 24.9 ± 5.2  23.1 (20.7 – 28.9) | 24.5 ± 3  24.3 (22.9 - 26) | > 0.05 |

Data are presented as median with IQR.

**Table S2.** Clinical data of CholU patients separated by sweating behavior. Significant differences between the groups were calculated using Kruskal-Wallis-Test or Chi-Square (*X²*) if indicated. p<0.05 is considered significant. A trend with p <0.1 is highlighted in **bold**.

| **Parameter** | **SRS** | **RS** | **NS** | **p** |
| --- | --- | --- | --- | --- |
| **Number** | 3 | 7 | 3 |  |
| **Sex (m/f)** | 2/1 | 6/1 | 1/2 | *X²* 0.27 |
| **Age (years)**  mean ± SD  median (IQR) | 37.7 ± 8.6  36 (30-47) | 31.9 ± 11  31 (20-37) | 33.3 ± 16.2  24 (24-52) | 0.73 |
| **Age at onset of disease**  mean ± SD  median (IQR) | 14.7 ± 6.8  17 (7-20) | 24.3 ± 10.9  22 (18-28) | 28.4 ± 11.9  23.2 (20-42) | 0.21 |
| **Persitance of disease**  mean ± SD  median (IQR) | ***23 ± 8.7***  ***27 (13-29)*** | ***7.5 ± 6.7***  ***5 (1.5-15)*** | ***8 ± 3.4***  ***10 (4-10)*** | ***0.09*** |
| **CholUSI**  mean ± SD  median (IQR) | 14.3 ± 2.9  16 (11-16) | 11.9 ± 2.3  12 (9-14) | 10.7 ± 0.6  11 (10-11) | 0.21 |
| **VAS disease severity**  mean ± SD  median (IQR) | 5.7 ± 3.5  5.6 (2.3-9.3) | N=6  4.1 ± 2.1  3.3 (2.4-6.3) | 5.8 ± 1.4  5.4 (4.6-7.3) | 0.69 |
| **Lickert disease severity**  mean ± SD  median (IQR) | 1.7 ± 1.2  1 (1-3) | N=6  2 ± 0.9  2 (1-3) | 2 ± 0  2 (2-2) | 0.7 |
| **CholUAS7**  mean ± SD  median (IQR) | 33.3 ± 43.9  10 (6-84) | 42 ± 36.2  46 (9-72) | 32 ± 8.5  32 (26-38) | 0.9 |
| **PCE: ProvoUAS**  mean ± SD  median (IQR) | 4 ± 1.7  5 (2-5) | 3.6 ± 1.7  3 (3-5) | 4.3 ± 1.2  5 (3-5) | 0.79 |
| **PCE: onset of sweating (min)**  mean ± SD  median (IQR) | 19.3 ± 4.6  22 (14-22) | 15.4 ± 3.3  15 (14-17) | 15.5 ± 3.5  15.5 (13-18) | 0.48 |
| **PCE: onset of whealing (min)**  mean ± SD  median (IQR) | 23.7 ± 3.1  23 (21-27) | 19.1 ± 3.2  19 (17-21) | 21.3 ± 3.2  20 (19-25) | 0.18 |
| **DLQI**  mean ± SD  median (IQR) | 11.3 ± 4.2  10 (8-16) | N=6  13.2 ± 6.9  12 (7.8-17) | 12 ± 3.6  11 (9-16) | 0.9 |
| **CholU-Qol**  mean ± SD  median (IQR) | 48 ± 15.5  47 (33-64) | N=6  50.8 ± 20.6  42 (36-74.3) | 51 ± 1  51 (50-52) | 0.76 |
| **UCT**  mean ± SD  median (IQR) | 8 ± 5.6  9 (2-13) | N=6  6 ± 3.7  6 (3.8-8.8) | 4.7 ± 1.2  4 (4-6) | 0.5 |
| **Erlangen Atopy score**  mean ± SD  median (IQR) | 7.3 ± 1.5  7 (6-9) | 7.1 ± 3.2  7 (4-10) | 6.3 ± 4  4 (4-11) | 0.9 |
| **Total IgE**  mean ± SD  median (IQR) | 391 ± 384  345 (33-796) | 123 ± 130  78 (22-286) | 147 ± 57  141 (93-207) | 0.32 |

IQR interquartile range

*X²* Chi-Square-Test

N number (given in table if less than total per column)

**Table S3**: Correlation of CHRM3 and ACh-E expression with clinical data. Correlations were calculated using spearman´s rank test. p<0.05 is considered significant and highlighted in bold.

|  | **AchE expression** | | **CHRM3 expression** | |
| --- | --- | --- | --- | --- |
|  | **Non-lesional** | **Lesional** | **Non-lesional** | **Lesional** |
| **Persistence of disease** | ***r = -0.69,***  ***p = 0.009 ***** | ***r = -0.77,***  ***p = 0.002 ***** | r = -0.37,  p > 0.05 | r = -0.41,  p > 0.05 |
| **CholUSI** | ***r = -0.63,***  ***p = 0.02 **** | ***r = -0.58,***  ***p = 0.04 **** | r = -0.42,  p > 0.05 | r = -0.25,  p > 0.05 |
| **Sweat grading** | ***r = 0.76,***  ***p = 0.004 ***** | ***r = 0.91, p=0.00006****** | ***r = 0.58,***  ***p = 0.049 **** | r = 0.42,  p > 0.05 |
| **VAS disease severity** | r = 0.01  p = 0.1 | r = 0.03  p = 0.9 | r = 0.21  p = 0.51 | r = -0.27  p = 0.43 |
| **Lickert disease severity**  **(** | r = 0.09  p = 0.8 | r = 0.02  p = 0.9 | r = 0.26  p = 0.38 | r = -0.31  p = 0.31 |
| **CholUAS7**  (points) | r = -0.13  p = 0.7 | r = -0.2  p = 0.6 | r = 0.33  p = 0.29 | r = -0.16  p = 0.6 |
| **PCE: ProvoUAS**  (points) | r = -0.01  p = 1 | r = 0.05  p = 0.9 | r = 0.3  p = 0.3 | r = 0.1  p = 0.7 |
| **PCE: onset of sweating**  (time,min) | r = -0.34  p = 0.26 | r = -0.28  p = 0.36 | ***r = -0.77***  ***p = 0.002*** | r = -0.41  p = 0.1 |
| **PCE: onset of whealing**  (time, min) | r = -0.61  p = 0.03 | ***r = -0.68***  ***p = 0.01*** | r = -0.4  p =0.18 | ***r = -0.61***  ***p = 0.03*** |
| **DLQI**  (points) | r = 0.02  p = 1 | r = 0.06  p = 0.86 | r = 0.3  p = 0.3 | r = -0.28  p = 0.38 |
| **CholU-Qol**  (points) | r = 0.1  p = 0.7 | r = 0.03  p = 0.9 | r = 0.25  p = 0.4 | r = -0.25  p = 0.47 |
| **UCT**  (points) | r = -0.3  p = 0.3 | r = -0.3  p = 0.3 | r = -0.25  p = 0.43 | r = 0.06  p = 0.8 |
| **Erlangen Atopy score**  (points) | r = 0.07  p = 0.8 | r = -0.15  p = 0.63 | r = -0.25  p = 0.4 | r = -0.14  p = 0.65 |
| **Total IgE**  (IU/ml) | r = -0.47  p = 0.1 | r = -0.14  p = 0.66 | r = -0.32  p = 0.29 | r = -0.35  p = 0.25 |
